# Low‐Level Alcohol Consumption in Children 11–12 Years Old Is Linked to Neural Substrates Involved in Reward Processing and Inhibitory Control: Results From a Brain‐Wide Association Study

**DOI:** 10.1111/acer.70370

**Published:** 2026-07-09

**Authors:** Isabella F. Jackson, Francisco A. C. Meyer, Ryan M. Sullivan, Ashley L. Watts

**Affiliations:** ^1^ Department of Psychological Sciences Vanderbilt University Nashville Tennessee USA; ^2^ Department of Psychiatry Yale School of Medicine New Haven Connecticut USA; ^3^ Department of Psychiatry University of California San Diego California USA

**Keywords:** adolescent development, alcohol use, neuroimaging

## Abstract

**Background:**

We comprehensively examined the cross‐sectional and prospective structural (i.e., cortical volume and subcortical volume) and functional (i.e., activation during the Monetary Incentive Delay task and the emotional N‐back task) neural correlates of alcohol sipping in early adolescence across two waves of data from the Adolescent Brain Cognitive Development (ABCD) Study (baseline: ages 9–10, *n* = 7555; 2‐year follow‐up: ages 11–12, *n* = 5892).

**Methods:**

Generalized linear mixed models examined individual brain regions of interests' ability to predict alcohol sipping.

**Results:**

At the 2‐year follow‐up, sipping was associated with activation during the MID large reward versus neutral contrast in 12 regions (e.g., nucleus accumbens), and activation during the MID large loss versus neutral contrast in 13 regions (e.g., insula).

**Conclusions:**

These findings aligned with existing literature on alcohol consumption, with neural regions involved in reward and inhibitory control being associated with alcohol sipping. Given that the findings from year 2 mirror several well‐established neural correlates of alcohol use and alcohol use disorder in adults, alcohol sipping may be an important phenotype for predicting future alcohol involvement.

## Graphical abstract

In adolescents aged 11–12 years, alcohol sipping was associated with activation in reward and inhibitory control related regions, including the nucleus accumbens, anterior cingulate cortex, and various regions of the prefrontal cortex.

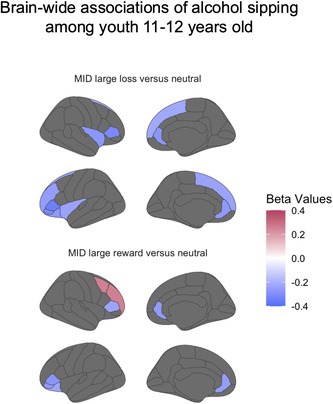

## 1 Introduction

In a 2019 survey, around 24.5% of adolescents aged 13–14 years old reported drinking alcohol, 10.1% reported experiencing drunkenness, and 3.8% reported binge drinking in the last 2 weeks (Johnston et al. 2020). Early alcohol exposure is concerning given that alcohol use during adolescence can have long‐lasting effects on physical and psychological health (Marshall 2014). For example, earlier alcohol initiation is associated with increased risk of later developing an alcohol use disorder (AUD) (DeWit et al. 2000), heavier use across the lifespan, and other psychosocial factors (e.g., parent drinking, peer alcohol initiation; Hawkins et al. 1997; Hingson et al. 2006).

### 1.1 Adolescent Alcohol Use and Its Neural Correlates

Adolescent alcohol use is also associated with differences in brain structure, function, and connectivity (Feldstein Ewing et al. 2014; Jacobus and Tapert 2013). Among adolescents, heavy alcohol use has been associated with smaller volume in the cerebellum, hippocampus, anterior cingulate cortex, inferior frontal gyrus, and cingulate gyrus (Lees et al. 2019; Squeglia, Jacobus, and Tapert 2014; Whelan et al. 2014). Among adults, heavy alcohol use has been associated with smaller volume in the prefrontal cortex, insula, nucleus accumbens, and amygdala (Makris et al. 2008).

Beyond brain structure, previous studies have yielded mixed results on the associations between adolescent alcohol consumption and functioning in two reward‐relevant regions of the brain, the ventral striatum and nucleus accumbens (Cope et al. 2019; Martz et al. 2022; Waller et al. 2019). Some evidence suggests that *stronger* nucleus accumbens activation during anticipation of monetary gain was predictive of substance use onset before age 16 (Cope et al. 2019), although other evidence suggests that children with a parental history of alcohol problems had *weaker* activation in the right nucleus accumbens and the ventral striatum during reward anticipation (Martz et al. 2022). Still, other evidence suggests that alcohol use was associated with *stronger* ventral striatum activation during reward anticipation at age 20, which in turn predicted alcohol consumption at age 22 (Waller et al. 2019). Nevertheless, a meta‐analysis of adult AUD patients found *weaker* ventral striatum activation during anticipation of monetary reward (Zeng et al. 2023). In addition to those reward regions, other studies have found weaker activation in the putamen, inferior frontal gyrus, and hippocampus in 14‐year‐old binge drinkers anticipating reward (Whelan et al. 2014).

Neural predictors of heavy alcohol use in adolescents also implicate executive function and emotion‐related processing (Lees et al. 2019). One study found that binge drinking in young adults was associated with stronger activation in the pre‐supplementary motor area, dorsomedial prefrontal cortex, cerebellum, thalamus, and insula during a working memory task, and stronger activation in the anterior cingulate cortex, inferior frontal gyrus, dorsolateral prefrontal cortex, and insula during an inhibitory control task (Pérez‐García et al. 2022). Although fewer studies have investigated the neural substrates potentially involved in both alcohol use and emotion regulation, one study showed that faster onset to adolescent binge drinking has been predicted by heightened activity in the nucleus accumbens during decision‐making tasks (Morales et al. 2018), another showed that young adult binge drinkers had weaker activation in the superior temporal gyrus and stronger activation in the middle frontal gyrus during an emotion recognition task (Maurage et al. 2013), and another found that teenage binge drinkers had weaker activity in the temporal pole and inferior frontal gyrus when processing angry faces (Whelan et al. 2014).

### 1.2 Targeting Earlier Indicators of Alcohol Initiation

Most research on early alcohol use focuses on teenagers (i.e., adolescents 13 and older), perhaps because younger adolescents do not typically drink in large quantities (Donovan and Molina 2008). A substantial portion of children report relatively low levels of alcohol consumption (Donovan and Molina 2013; Lisdahl et al. 2021; Watts et al. 2021), with 23% of the Adolescent Brain Cognitive Development Study cohort (*N* = 11,878) reporting sipping alcohol by age 9 or 10 and 17% reporting that such sipping occurred outside of a religious context (Lisdahl et al. 2021; Watts et al. 2021). Alcohol sipping is, in turn, prospectively predictive of finishing a full drink, getting drunk, and engaging in heavy drinking by age 14, indicating that alcohol sipping may reflect an early emerging phenotype that forecasts risk for heavier drinking (Jackson et al. 2015). The potential compatibility between early alcohol sipping and teenage alcohol use motivates the extension of studying the neural risk for alcohol initiation to youths.

Few studies have examined the structural and functional neural correlates of alcohol sipping in early adolescence (i.e., ages 10–12), and they have tended to focus on only one or two brain regions that are defined in an a priori manner in confirmatory investigations. One study found no relationship between sipping and functional activation in the nucleus accumbens and inferior frontal gyrus during a reward task (May et al. 2022), and another found no relationship between sipping and functional connectivity between the nucleus accumbens and the frontoparietal network (Harris et al. 2023). Another study found that activation in the dorsal anterior cingulate cortex during an inhibitory control task moderated the relationship between sipping and both personality and depression (Ferariu et al. 2024).

### 1.3 Present Study

We aimed to address the limited literature by comprehensively examining the structural and functional neural correlates of alcohol sipping via a brain‐wide association study (Marek et al. 2022) using two waves of the Adolescent Brain and Cognitive Development (ABCD) Study, a longitudinal sample of 9–10 year‐olds (*N* = 11,872, 53% female; Garavan et al. 2018). Although confirmatory investigations are often prioritized in the literature, brain‐wide association studies add much in the way of value above and beyond confirmatory ones because they are data‐driven and can be particularly useful to reveal unexpected or novel associations that might have been overlooked in targeted confirmatory approaches, particularly for research questions where the neural mechanisms of a phenotype are not well understood. Relatedly, because the field increasingly appreciates the fact that brain regions do not operate in silos, brain‐wide analyses lead to a better appreciation of the distributed and interconnected nature of neural processes involved in alcohol sipping, which can lead to hypothesis generation and theory development for future confirmatory studies.

In terms of brain structure, we focused on cortical and subcortical volume. Cortical volume provides a comprehensive measure, as it is the product of cortical thickness and surface area. Examining cortical volume also facilitates comparison with existing literature (Cheetham et al. 2014; Lees et al. 2019; Squeglia, Rinker, et al. 2014), where cortical volume has been commonly examined across alcohol‐related phenotypes. In terms of brain functioning, we examined activation during tasks that elicit reward processing and emotion regulation (i.e., the momentary incentive delay task [large reward > neutral, large loss > neutral], the emotional N‐back task [happy > neutral, fearful > neutral faces]), two cognitive processes posited to play a role in alcohol sipping and related phenotypes (e.g., Watts et al. 2021).

## 2 Methods

### 2.1 Participants

The ABCD study follows a sample of 11,872 US‐based youths aged 9–10 years old across development within 21 US‐based catchment sites (Garavan et al. 2018). Participants were required to be fluent in English and have no history of severe mental illness, autism spectrum disorder, intellectual disability, major neurological disorder, or traumatic brain injury. Data are publicly available through the National Institute for Mental Health Data Archive (https://nda.nih.gov/). Our project's OSF page contains all code necessary to fully reproduce our findings upon acquisition of the ABCD data (https://doi.org/10.17605/OSF.IO/NSHFB).

We focused on the subset of subjects who passed quality control for the structural and functional MRI scans and did not have missing alcohol use data (baseline: *n* = 7555; 2‐year follow‐up: *n* = 5892). At baseline, 49% of participants were female, and 66% of participants identified as White, 19% as Hispanic, 17% as Black, 7% as Asian, and 10% as Other.

### 2.2 Alcohol Sipping Assessment

Alcohol sipping was measured annually with the 10‐item self‐reported iSay Sip Inventory (Jackson et al. 2015). We focused on the number of occurrences that youths reported sipping alcohol outside of a religious context. Counts reflected the number of lifetime occasions at baseline and the number of occasions since the last assessment after baseline. Given that neuroimaging data were not assessed at the year 1 follow‐up, we combined the number of sipping occurrences reported at year 1 and year 2 follow‐ups (i.e., since baseline) to carry them into the year 2 analyses.

### 2.3 Monetary Incentive Delay Task

The Monetary Incentive Delay (MID) task intends to measure reward responsivity during the anticipation and receipt of loss or reward (Knutson et al. 2000). Participants receive incentive cues that indicate gaining or losing a potential reward (e.g., 20 cents, $5) then respond to a target to win or avoid losing money. They also receive neutral cues (e.g., $0). Cues are followed by a delay, then a target period during which the participant must respond by pressing a button to win or avoid losing the money (see Casey et al. 2018, for additional details). Given the focus on reward process in adolescent alcohol research, we focused on the contrasts between (1) anticipation of a large reward versus neutral (i.e., gaining $5 vs. $0) and (2) anticipation of a large loss versus neutral (i.e., losing $5 vs. $0).

### 2.4 Emotional N‐Back Task

The emotional N‐back (EN‐back) task intends to measure working memory in addition to implicit emotion regulation and reactivity (Cohen et al. 2016). It uses a high and a low load memory condition (i.e., 2‐back vs. 0‐back) and includes happy, fearful, neutral faces, and places as stimuli. For the 2‐back condition, participants are instructed to indicate the current stimuli as a “match” if it is the same stimuli from two trials before the current trial. For the 0‐back condition, participants are instructed to indicate if the current stimuli is a “match” for the first stimuli shown at the start of the run (Casey et al. 2018). Given our interest in emotion regulation, we focused on the contrasts between (1) happy and neutral faces and (2) fearful and neutral faces.

### 2.5 Neuroimaging

Scans were conducted using either a GE MR750, Siemens PRISMA, or Philips scanner using a 32‐channel head coil. A 3D T1‐weighted magnetization‐prepared rapid acquisition gradient‐echo scan using prospective motion correction (PROMO) is used to obtain a high‐resolution anatomical image for cortical and subcortical segmentation (see Hagler Jr et al. 2019, for more details).

The ABCD Data Analytics and Informatics Resource Center processes imaging data (Hagler Jr et al. 2019). Structural and functional regions of interest were defined using the Desikan cortical parcellation atlas (Desikan et al. 2006), and subcortical areas were segmented using the Fischl subcortical atlas (Fischl et al. 2002). Of the cortical atlases provided in the ABCD study, the Desikan atlas provides the most sensible compromise between number of ROIs and statistical power. Additionally, several anatomical and functional task effects have been demonstrated at this level of parcellation in the ABCD cohort (Gonçalves et al. 2024; Lees et al. 2020). Structural metrics of interest included cortical volume, whereas functional variables included beta contrasts between blood‐oxygenated level‐dependent (BOLD) hemodynamic response during the MID and EN‐back tasks (e.g., happy > neutral faces). Structural measures are based on averages within a region and functional measures are averaged within a region and across two runs for each task. Most subcortical regions were included in the subcortical volume and functional analyses, but some regions (i.e., cerebellar white matter, cerebral white matter, inferior lateral ventricle, lateral ventricle, third and fourth ventricles) were excluded due to convergence issues.

### 2.6 Data Analysis

We performed all analyses using the R statistical program version 4.4.1 (R Core Team 2024) with the lme4 (Bates et al. 2015), lsmeans (Lenth 2016), and parameter (Lüdecke et al. 2020) packages. We tested individual brain region of interests' (ROI) ability to predict alcohol sipping for the baseline and year 2 data with negative binomial regressions to account for the count distribution of sipping in a generalized linear mixed modeling framework. We used the following R model specification: sip_count ~ scale(roi) + scale(interview_age) + factor(demo_sex_v2) + (1 | mri_info_deviceserialnumber) + (1 | rel_family_id). We also tested baseline brain ROIs ability to predict year 2 sipping across all ROIs.

To do so, we regressed each ROI onto the sipping count and included fixed effects for age and sex and random effects for the family identification variable (some ABCD participants were siblings) and the scanner serial number. The inclusion of these random effects properly accounts for the nonindependence of observations across scanners and within families. Given the number of tests conducted, we adjusted all *p* values within each analysis type (e.g., cortical volume, MID large reward > neutral) using false discovery rate. We further used an alpha threshold of 0.001 to guard against false positives given the large size of the ABCD study (see Tables S1–S10) for all unadjusted and adjusted *p* values; these tables also include *p* values using Bonferroni correction. Beta coefficients for all ROIs can be interpreted as the log expected count for a 1‐unit increase in ROI volume or activation (depending on the analysis). The beta coefficients can be exponentiated to produce the multiplicative effect of the ROI on sipping, known as the incident rate ratio.

For our cross‐sectional analyses at year 2, in addition to the sipping since baseline assessment, we also considered lifetime sipping's association with year 2 brain structure and function (Tables S1–S10). The pattern of significant ROIs in those results reflect a combination of the findings unique to each the baseline and year 2 waves described in the results. As such, in combination with the fact that sipping was unstable across the waves, we focused on since last assessment sipping at year 2 so as not to conflate baseline and year 2 associations.

## 3 Results

### 3.1 Sipping Descriptives

At baseline, 18% of youth in our sample reported sipping alcohol, with an average of 2.67 lifetime sipping occasions (range: 1–158). At year 2, only 11% of youth in our sample reported sipping since the baseline assessment, with an average of 3.43 sipping occasions (range: 1–60).

### 3.2 Structural Correlates

#### 3.2.1 Baseline

##### 3.2.1.1 Cortical

**FIGURE 1:**
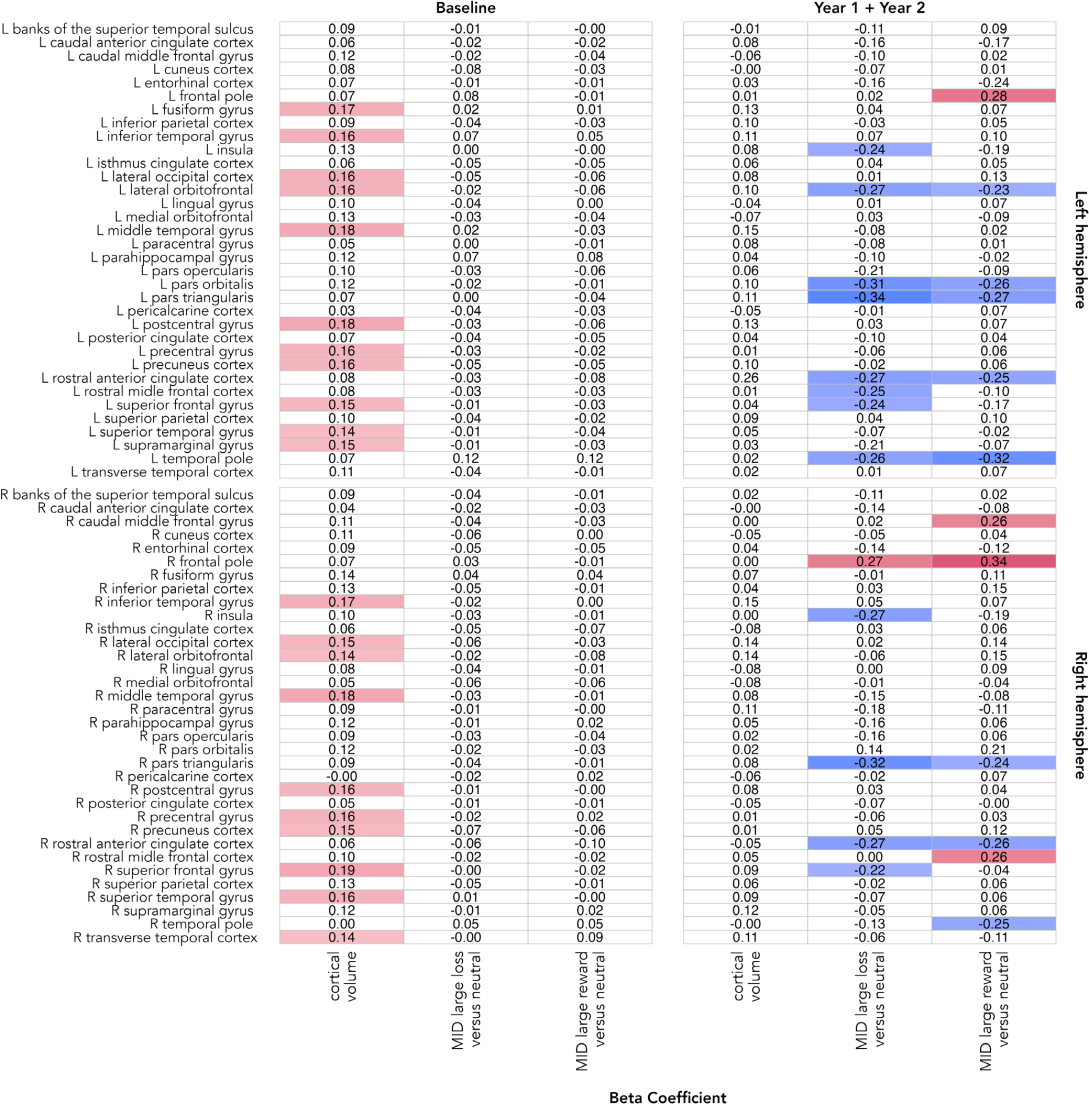
Heatmap of beta coefficients between alcohol sipping and cortical regions across waves. All beta coefficients are displayed. The boxes are colored either pink or purple when the false discovery rate adjusted *p* value < 0.001. Pink boxes indicate negative associations, and purple boxes indicate positive associations.

**FIGURE 2:**
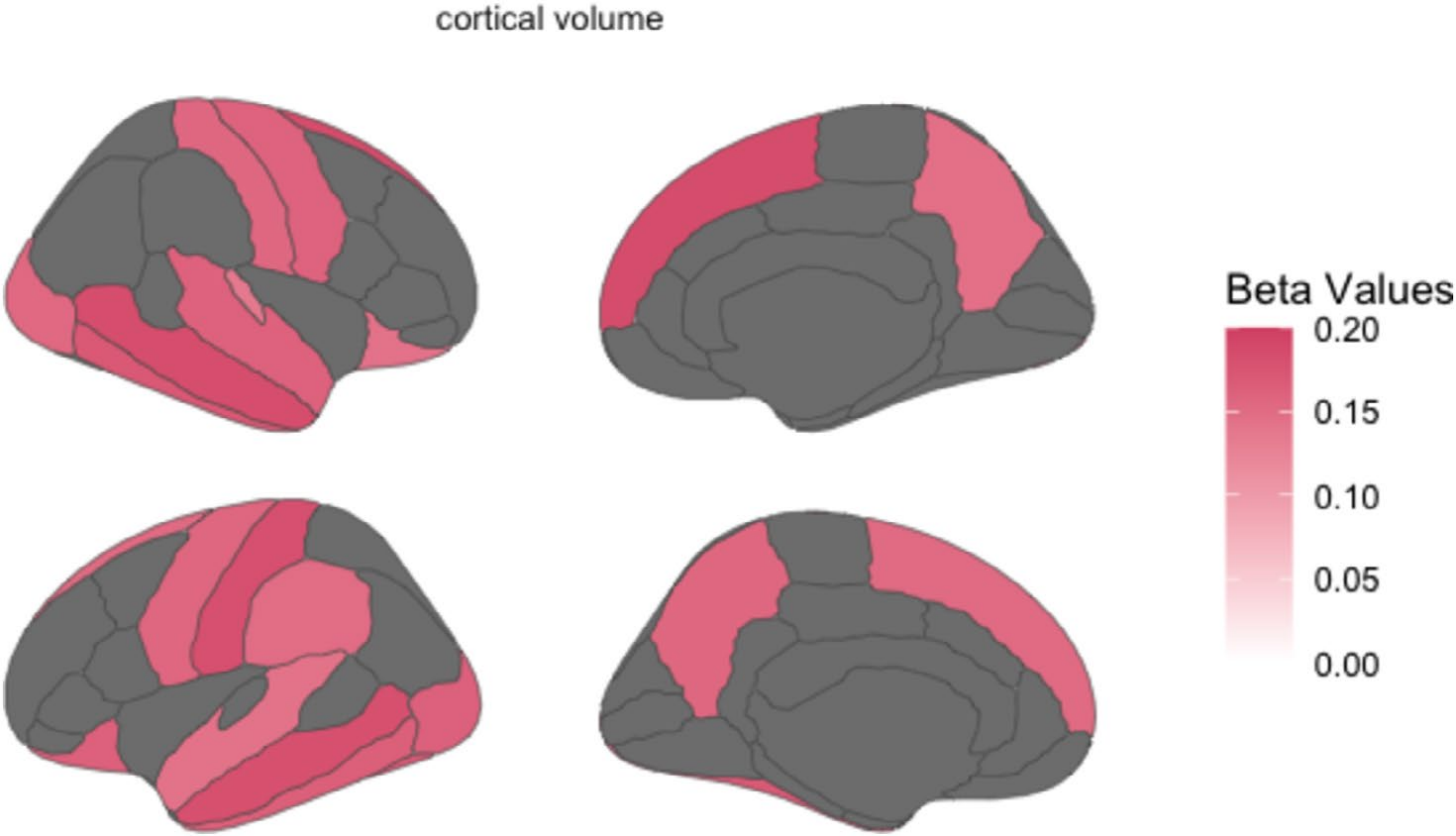
Cortical volume and associations with alcohol sipping at baseline. The temporal and frontal poles are not visible in this figure.

Baseline alcohol sipping was associated with larger cortical volume in 21 of the 68 ROIs in the Desikan atlas (Bs ranged from 0.14 to 0.19), although the magnitudes of these associations were generally quite small (Figures 1 and 2). The largest effect size was for the right superior frontal gyrus (B = 0.19). Cortical volume for these ROIs was not associated with baseline sipping after covarying total intracranial volume (Bs ranged from −0.02 to 0.07; Table S1).

##### 3.2.1.2 Subcortical

**FIGURE 3:**
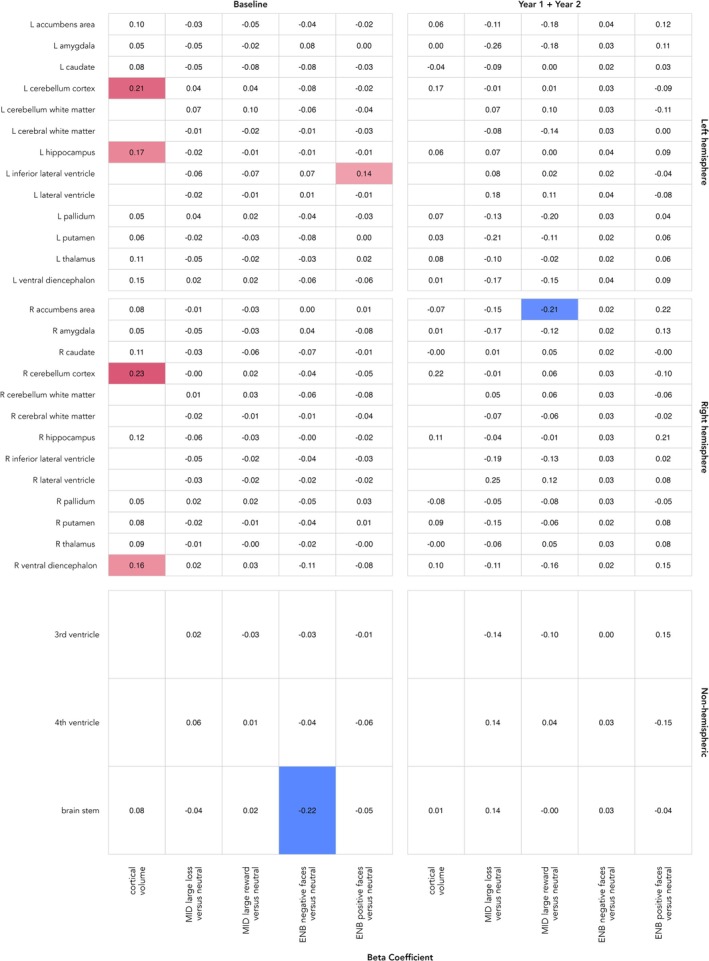
Heatmap of beta coefficients between alcohol sipping and for subcortical regions across waves. All beta coefficients are displayed. The boxes are colored either pink or purple when the false discovery rate adjusted *p* value < 0.001. Pink boxes indicate negative associations, and purple boxes indicate positive associations. Blank boxes indicate ROIs for which the models did not converge during analysis.

**FIGURE 4:**
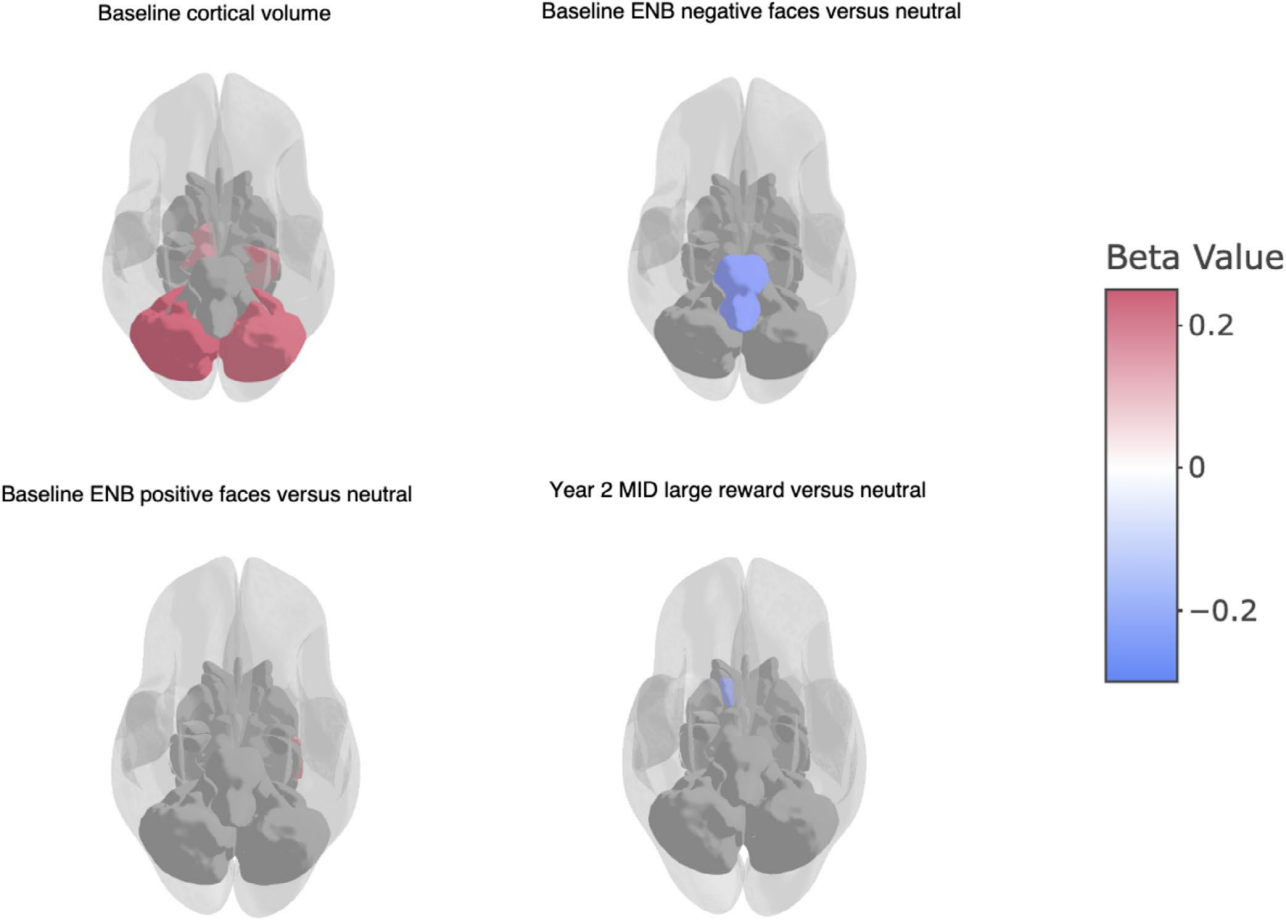
Subcortical structural and functional correlates of alcohol sipping at baseline and the 2‐year follow‐up.

Alcohol sipping was associated with larger volume in 4 of the 30 subcortical ROIs in the automatic segmentation atlas (Bs ranged from 0.16 to 0.23; Figures 3 and 4): the left and right cerebellum cortex, left hippocampus, and right ventral diencephalon. The largest effect sizes were for the left and right cerebellum cortices (Bs were 0.21 and 0.23, respectively). Cortical volume for these ROIs was not associated with baseline sipping after covarying total intracranial volume (Bs ranged from 0.01 to 0.16; Table S6).

#### 3.2.2 Year 2

##### 3.2.2.1 Cortical

Year two alcohol sipping was not associated with the cortical volume of any ROIs (Table S1).

##### 3.2.2.2 Subcortical

Sipping was not associated with subcortical volume at year 2 (Table S6).

### 3.3 Functional Correlates

#### 3.3.1 Baseline

##### 3.3.1.1 Cortical

Sipping was not associated with any cortical ROIs for the MID task or for the EN‐back task at baseline (Tables S2–S5).

##### 3.3.1.2 Subcortical

Sipping was associated with the weaker activation in the brainstem (B = −0.22) for the EN‐back negative face versus neutral contrast and stronger activation in the left inferior lateral ventricle (B = 0.14) for the EN‐back positive face versus neutral contrast (Figures 3 and 4).

#### 3.3.2 Year 2

##### 3.3.2.1 Cortical

**FIGURE 5:**
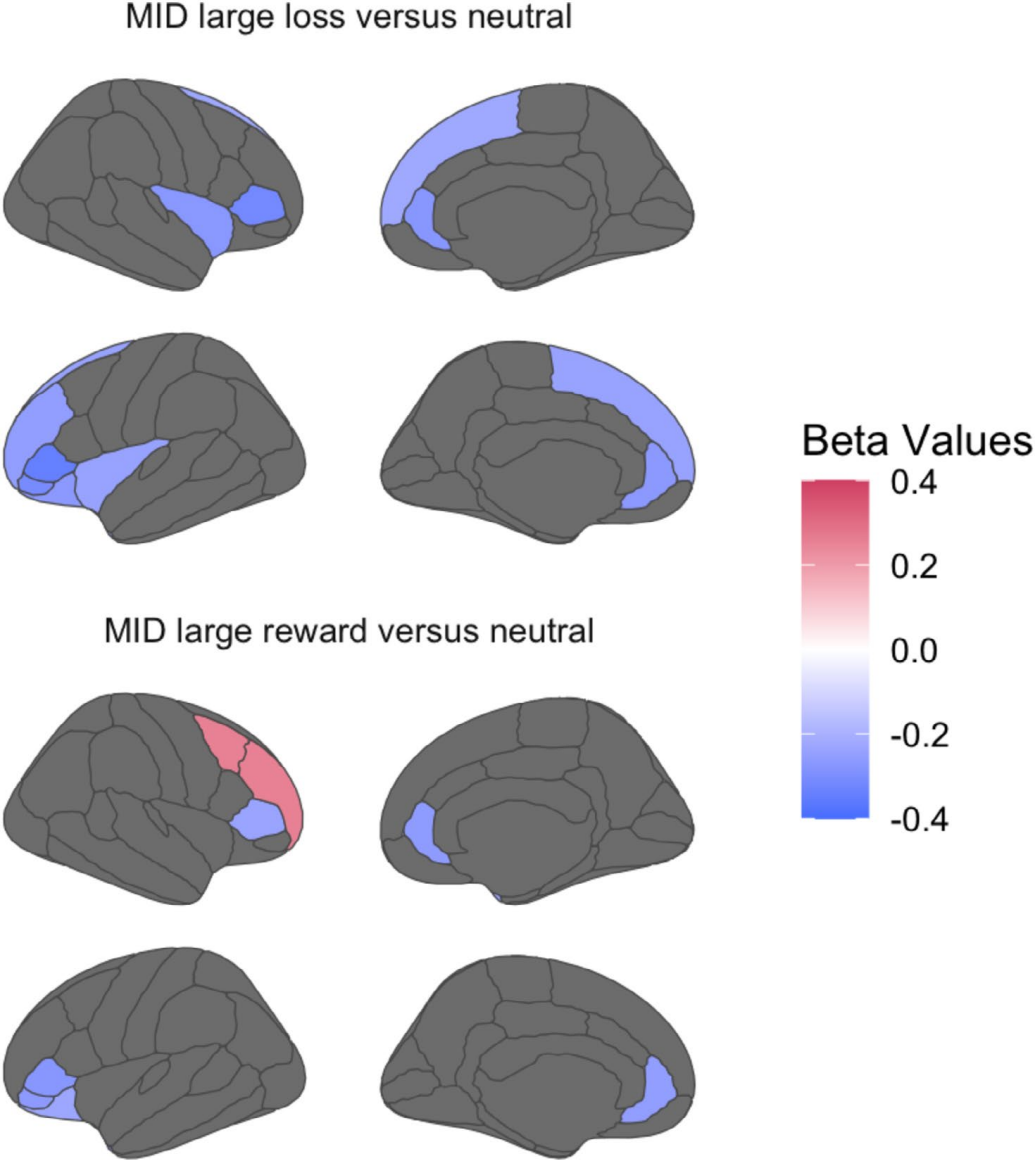
Cortical functional correlates of alcohol sipping at the 2‐year follow‐up. The temporal and frontal poles are not visible in this figure.

Of the 68 cortical ROIs, alcohol sipping was associated with 13 ROIs in the MID large loss versus neutral contrast (Bs ranged from −0.34 to 0.27) and 12 ROIs in the MID large reward versus neutral contrast (Bs ranged from −0.32 to 0.34). A majority of these ROIs were located in the prefrontal cortex (Figures 1 and 5). Activation in most of these ROIs was not associated with year 2 sipping after covarying baseline sipping (Bs ranged from −0.11 to 0.20; Tables S15 and S16). Only the association for the right front pole in the MID large reward versus neutral contrast survived after covarying baseline sipping (B = 0.28). Sipping was not associated with activation in any cortical ROIs for the EN‐back task (Tables S4 and S5).

##### 3.3.2.2 Subcortical

Sipping was negatively associated with activation in the right accumbens (B = −0.21) for the MID large reward versus neutral contrast (Figures 3 and 4). Activation in the right accumbens was not associated with year 2 sipping after covarying baseline sipping (Bs ranged from −0.12 to 0.11; Tables S20 and S21). Sipping was not associated with activation in any subcortical ROIs for the EN‐back task (Tables S9 and S10).

### 3.4 Baseline Brain Structure and Functioning Predicting Year 2 Sipping

There were no prospective associations between baseline brain volume or activation and sipping across all structural and functional analyses (Tables S24–S33).

### 3.5 Replication Across Waves

Consistency in the associations between alcohol sipping and brain volume and function across the two waves was low (intraclass correlation coefficient, or ICC [2,1] = 0.13 [95% CI: 0.04, 0.22]). Potentially owing to the poor replicability in the neural correlates of sipping across waves, sipping was largely unstable from baseline (i.e., lifetime number of sips at baseline) to year 2 (i.e., number of sips since baseline; ICC [2,1], = 0.11 [95% CI: 0.09, 0.13]). This inconsistency across waves may reflect the poor consistency observed in task‐based fMRI more generally (Herting et al. 2018).

The stability of brain structure and function across waves varied depending on the measure. Generally, the ICCs for structural brain volume ROIs were high across the cortical and subcortical measures (ICCs ranged from 0.89 [subcortical volume] to 0.92 [cortical volume]). In contrast, the ICCs for functional brain activation were low (ICCs ranged from 0.00 [cortical ENB negative face versus neutral contrast, subcortical ENB positive face versus neutral contrast, ENB negative face versus neutral contrast] to 0.07 [cortical MID large reward vs. neutral contrast]; Table 1). We also considered the role of attrition in the lack of replication of our findings between baseline and the 2‐year follow‐up. 5892 participants from baseline (78%) completed the 2‐year follow‐up neuroimaging assessment, passed quality control, and reported on their alcohol consumption.

**TABLE 1:** Replication across waves.

Variable	ICC (2,1)	LL CI	UL CI	SD	Min	Max
Beta coefficients	0.13	0.04	0.22	—	—	—
Alcohol sipping	0.11	0.09	0.13	—	—	—
Cortical structural volume	0.92	—	—	0.05	0.76	0.97
Cortical MID large reward vs. neutral contrast	0.07	—	—	0.04	0.00	0.19
Cortical MID large loss vs. neutral contrast	0.04	—	—	0.04	0.00	0.17
Cortical ENB positive faces vs. neutral contrast	0.01	—	—	0.01	0.00	0.04
Cortical ENB negative faces vs. neutral contrast	0.00	—	—	0.00	0.00	0.02
Subcortical structural volume	0.89	—	—	0.07	0.73	0.97
Subcortical MID large reward vs. neutral contrast	0.04	—	—	0.03	0.00	0.09
Subcortical MID large loss vs. neutral contrast	0.03	—	—	0.02	0.00	0.06
Subcortical ENB positive faces vs. neutral contrast	0.01	—	—	0.01	0.00	0.05
Subcortical ENB negative faces vs. neutral contrast	0.00	—	—	0.01	0.00	0.03

*Note:* To consider replication across waves, we computed ICCs of a variety of variables at baseline and the 2‐year follow‐up. ICCs for beta coefficients quantified the congruence between betas reflecting the association between alcohol sipping and all ROIs across time for all neuroimaging indices (e.g., cortical structural volume, the cortical momentary incentive delay contrast for large reward vs. neutral). ICCs for alcohol sipping considered the congruence of the sipping counts across time. For all neuroimaging indices, we computed ICCs between each ROI for each neuroimaging index (e.g., cortical volume, the subcortical emotional N‐back contrast between positive and neutral faces). Given the number of ROIs, we summarize the mean ICCs for neuroimaging indices, along with the standard deviation, minimum, and maximum of the ICCs. Tables S11 and S12 contain all ICCs for individual ROIs.

Abbreviations: ENB, emotional N‐back task; ICC, intraclass correlation coefficient; LL CI, lower limit of the 95% confidence interval; MID, momentary incentive delay task; UL CI, upper limit of the 95% confidence interval.

To characterize the effects of attrition on our results, we took two steps. First, we re‐ran our baseline analyses using only the subset of data that completed the 2‐year follow‐up and computed ICCs (2,1) between the beta coefficients across all ROIs and neuroimaging indices. Replication was strong, albeit far from perfect (ICC [2,1] = 0.75 [95% CI = 0.71, 0.79], df = 474). Second, we computed ICCs between the beta coefficients across all ROIs and neuroimaging indices using the subset of participants at baseline who completed the 2‐year follow‐up and the 2‐year follow‐up data. Replication was extremely weak and near zero (ICC [2,1] = 0.02 [95% CI = −0.07, 0.11], df = 474). Taken together, these findings suggest that the lack of replication between baseline and the 2‐year follow‐up was attributable to genuine differences between the waves, as opposed to attrition.

### 3.6 Neuroimaging Inclusion/Exclusion

To consider the role of missingness in our analyses, we conducted logistic regressions that examined the relationship between sipping and having viable neuroimaging data. Sipping was not associated (*p* > 0.05, Table S13) with having available T1‐weighted structural data, MID task data, and EN‐back task data. Thus, excluded neuroimaging data had little to no statistical impact on our results.

## 4 Discussion

Early alcohol sipping is a potentially important indicator of future alcohol‐related behaviors. In this brain‐wide association study, we aimed to identify structural and functional brain correlates of alcohol sipping across childhood development when youths were ages 9–10 and ages 11–12. Although we identified several neural correlates of sipping, no significant neural correlates replicated across the waves. That is, we identified nonoverlapping sets of neural correlates at baseline and the 2‐year follow‐up. Given the strong consistency in structural brain volume across the waves, the lack of replicability in the sipping‐brain associations across waves was probably attributable to the instability in the sipping phenotype across time. Lack of replication across waves for the functional neural correlates was likely also driven by the instability in activation during the laboratory tasks across waves.

Most of the neural correlates of sipping at baseline were small, restricted to structural volume, and were largely inconsistent with the existing literature. These findings also did not retain significance after controlling for total intracranial volume. In contrast, the findings from the 2‐year follow‐up were generally more consistent with the literature. Given that sipping was unstable across the two waves, we suspect that there is a developmental shift in alcohol sipping whereby the 2‐year follow‐up is more reflective of a phenotype that forecasts risk for alcohol involvement across the lifespan. Supporting such a conclusion, average alcohol consumption was more frequent at the 2‐year follow‐up than at baseline. As such, we focus much of our discussion on the 2‐year follow‐up findings.

### 4.1 Consistencies With the Existing Literature

Three limbic system regions—the rostral anterior cingulate cortex (rACC), nucleus accumbens (NAcc), and insula—were associated with alcohol sipping at year 2 but in different directions. Sipping was associated with weaker (or blunted) activation in the left and right rACC during anticipation of both large losses and large rewards. That is, alcohol sipping was linked to weaker activation in the left rACC during anticipation of monetary outcomes, including losses and gains. The rACC is known to play a role in reward processing, decision‐making, and cognitive control, with stronger rACC activity indicating reward sensitivity (Whittle et al. 2006). As such, this finding is relatively inconsistent with speculation that early alcohol initiation reflects reward sensitivity (e.g., Watts et al. 2021, 2024).

We also found that alcohol sipping was associated with weaker activation in the right NAcc for the large reward versus neutral contrast. This finding contradicts previous work that showed that substance use initiation was associated with stronger activation during monetary reward anticipation (Cope et al. 2019). In contrast, our finding aligns with other studies that have found weaker activation in the right NAcc for children with a family history of alcohol problems during reward anticipation (Martz et al. 2022). Nevertheless, other family history studies have also found that children with a parental history of alcohol problems had more activation in the right NAcc relative to controls (Kwarteng et al. 2021). General alcohol‐related vulnerabilities—whether genetic, or due to early experimentation, or both—appear to have mixed findings associated with activation in the right NAcc, at least in children. Studies assessing children with family alcohol history may examine a phenotype that is distinct from alcohol use, which would limit their comparability.

Regarding other limbic regions, alcohol sipping was also associated with weaker activation in the left and right insula during anticipation of large losses during the MID task. The insula is implicated in the processing of disgust, anger, and anxiety, as well as cognitive control (Gasquoine 2014). Weaker activation in the insula during anticipation of large losses may suggest impaired or blunted neural mechanisms for assessing the salience and potential consequences of risky outcomes (Uddin 2015). Existing research also supports the role of the insula in alcohol consumption. For instance, one study found that sensation‐seeking was associated with hypoactivation of the insula during the absence of reward among adolescents (Cservenka et al. 2013). With this finding in mind, alcohol sipping may be related to insular activity by way of sensation seeking, which aligns with sensation seeking in the ABCD cohort (Watts et al. 2021, 2024).

Alcohol sipping was also associated with weaker activation of the left lateral orbitofrontal cortex during reward and loss anticipation. Of the prefrontal cortex correlates of alcohol sipping that we identified, the lateral orbitofrontal cortex was most consistent with the literature. The lateral orbitofrontal cortex is widely understood to be involved in the processing of information relevant to reward and punishment, the evaluation of the salience and valence of potential outcomes (Rolls et al. 2020), and impulse control and the ability to adjust behavior based on changing contingencies (Hooker and Knight 2006). Weaker activation in the lateral orbitofrontal cortex during the anticipation of both rewards and losses in individuals who consume larger amounts of alcohol may indicate impairment in neural processes that support the appropriate evaluation and representation of the motivational significance of potential outcomes, contributing to risk‐taking behavior. In other work, weaker activation in the orbitofrontal cortex was also correlated with greater drinking problems and urgency scores in older adolescent binge drinkers (Pérez‐García et al. 2022).

Also, two of the three prefrontal cortex structures that make up the inferior frontal gyrus were associated with sipping: the pars orbitalis and the pars triangularis. These regions are involved in cognitive control, response inhibition, and decision‐making (Swick et al. 2008). Alcohol sipping was associated with weaker activation in the left and right pars triangularis and the left pars orbitalis during both reward and loss anticipation. Weaker activation in these regions may indicate impairment in cognitive control and decision‐making among youths who consume larger amounts of alcohol. A previous study found similar results in 14‐year‐old binge drinkers with reduced activity in the inferior frontal gyrus during both reward anticipation and emotion processing tasks (Whelan et al. 2014).

Other factors such as genetic liability, family history of AUD, and early environmental stressors have been shown to contribute to neural vulnerability for alcohol use (Kirsch et al. 2020; O'Halloran et al. 2017). For instance, several studies have found structural and functional differences in brain regions implicated in reward, inhibitory control, and emotion processing for family history positive youth (Cservenka 2016; Cservenka and Azma 2025). These differences have been observed in youth with minimal alcohol exposure, suggesting that they reflect premorbid vulnerabilities. In fact, neural correlates of alcohol use more broadly often reflect predisposing factors (Baranger et al. 2023).

### 4.2 Underexplored Correlates of Alcohol Initiation

In addition to the neural regions that are well studied in the alcohol literature, our brain‐wide association study revealed underexplored correlates that warrant further consideration. Alcohol sipping was associated with weaker activation in the right temporal pole during reward anticipation and the left temporal pole during both reward and loss anticipation. The temporal pole plays a broad role in various aspects of cognition such as autobiographic memory, language, visual cognition, and social function (Herlin et al. 2021). In contrast to our findings, temporal pole activation has been associated with concurrent alcohol use during tasks involved in emotion processing, but not reward (Whelan et al. 2014). Nevertheless, prospective analyses have suggested that temporal pole activity during reward anticipation may be predictive of future alcohol use (Whelan et al. 2014). The role of the temporal pole in alcohol use requires further study, making it a potentially intriguing target to consider in future research.

As interesting as the temporal pole may be in terms of its relevance to early alcohol initiation, it is also possible that these findings reflect Type I errors. Brain‐wide association studies can uncover novel relationships between alcohol use and neural regions that are not widely targeted in the literature (Marek et al. 2022). At the same time, with the large number of statistical tests involved in brain‐wide association studies, there is an increased risk of false positive findings, which can lead to spurious conclusions (Marek et al. 2022). We adopted more stringent statistical corrections and thresholds (our *p* values were adjusted according to the false discovery rate, and we further adopted a more stringent alpha threshold of 0.001) to guard against this concern, but we cannot conclude firmly that each of our statistically significant associations between alcohol sipping and brain regions will replicate in other data or across time, meaning that they may not bear serious implications for the neurobiology of alcohol involvement. With neural regions whose functional roles are not always immediately clear, some findings are challenging to interpret but potentially worthwhile to pursue in replication efforts.

One such example was our reward anticipation findings for several regions of the prefrontal cortex, such as the rostral middle frontal gyrus, caudal middle frontal gyrus, superior frontal gyrus, and the frontal pole. Although each of these regions is relatively widely studied in the broader reward processing literature (Dichter et al. 2012; Zhang et al. 2013), they have been less frequently implicated in reward‐related processes within the alcohol literature. Moreover, the existing literature further finds that the middle frontal gyrus is more implicated in reward receipt than anticipation (Haber and Knutson 2010). In the context of alcohol research, the middle frontal gyrus and superior frontal gyrus tend to be associated with inhibitory control, emotion recognition, and working memory tasks rather than reward processing (Feldstein Ewing et al. 2014; Maurage et al. 2013), suggesting that further work is needed to clarify its possible role in alcohol use. Another example that stands out as a potentially spurious finding is the observed blunted brain stem activation during the EN‐back task due to the little existing research on this region being engaged during working memory or emotion‐related tasks (per Yarkoni et al. 2011).

### 4.3 Limitations and Future Directions

Our study possesses several important limitations. First, the rate of attrition between the baseline and year 2 sample for participants with available alcohol use and fMRI data was 22%, which may unduly influence the generalizability of our findings. Second, the structure of the sipping assessment at baseline and the 2‐year follow‐up differed, which complicates the comparison of sipping across development. At baseline, the assessment queries the number of sipping occasions in one's lifetime. At the 2‐year follow‐up, the assessment queries the number of occasions since the last interview. As a result, the differing assessment structure generates different distributions of sipping across time. Baseline sipping appeared more frequent than subsequent sipping because it was assessed over a much larger window of time, even though the average number of sipping occasions increased across waves.

Third, although the neural correlates of sipping at the 2‐year follow‐up were modestly compatible with the existing literature, it will be important to demonstrate in the ABCD study that current levels of sipping are prospectively associated with heavier use and related phenotypes (Jackson et al. 2015). The youths in the ABCD study were not, on balance, engaging in heavy drinking by the 2‐year follow‐up. Additional follow‐up assessments will allow us to examine whether neural correlates of alcohol sipping are indeed compatible with the neural correlates of heavier use.

Fourth and relatedly, this current data release for the ABCD Study precludes informing richer developmental questions surrounding the neural correlates of alcohol involvement. Modeling trajectories of alcohol use and brain development requires three or more waves of data, and only two waves of neuroimaging data are currently available. As the ABCD study expands and the youths age, we anticipate that developmentally focused research questions will be necessary to fully inform the neural correlates of early alcohol initiation. One such research avenue includes examining the relationship between trajectories of alcohol sipping and brain development. Such efforts may yield even more detailed insights into the nature of the relationship between alcohol use and brain structure and function, including whether neural correlates reflect premorbid risk for alcohol involvement or are due to neurotoxic effects of alcohol (Baranger et al. 2023).

Fifth, we did not examine sex differences in the neural correlates of alcohol sipping in the present study. Prior work has shown that associations between family history of alcohol use and brain development may be more pronounced among male youth (Gonçalves et al. 2024), whereas other findings indicate that neural risk for heavy drinking may be more pronounced among adolescent girls (Seo et al. 2019). Given the influence of sex on neurodevelopment (Vijayakumar et al. 2016), future work should investigate whether there are sex‐specific neural correlates of early alcohol use.

Finally, future research should consider using other modalities of operationalizing brain structure and function, such as task (or context‐dependent) and resting state functional connectivity. Given that brain regions rarely, if ever, operate in silos, consideration of the correlated activity among anatomically distinct brain regions is better suited to inform how the brain operates as a coordinated, dynamic system (Van Den Heuvel and Hulshoff Pol 2010). Relatively little research has considered the relationship between early alcohol use and functional connectivity, though some research suggests that youth at risk for alcohol problems have altered connectivity patterns, particularly in regions implicated in executive functioning, reward, and emotion processing (Nguyen‐Louie et al. 2018). Nevertheless, most existing studies are based on relatively small samples that are arguably underpowered to detect replicable group differences (Marek et al. 2022), making the ABCD Study data a rich landscape for exploring the neural correlates of early alcohol involvement.

## 5 Conclusion

The large size of the ABCD Study provides among the best powered examinations of the neural correlates of early alcohol use to date (Marek et al. 2022). This consideration is especially important given the relatively low base rate of alcohol use among youth. Brain‐wide association studies create the opportunity to identify novel neural correlates beyond those detected in confirmatory investigations, and our findings provide a comprehensive account of the structural and functional correlates of alcohol sipping in childhood.

Alcohol sipping in children is generally regarded as a developmental precursor to heavier drinking and has been associated with sensation‐seeking, fun‐seeking, and impulsivity (Jackson et al. 2015; Watts et al. 2021, 2024). We have expanded this literature on alcohol sipping by identifying previously studied and novel structural and functional neural correlates of sipping, especially through functional correlates relevant to reward processing. Our findings support the connection between alcohol involvement and reward and inhibitory control related regions, including the nucleus accumbens, anterior cingulate cortex, and various regions in the prefrontal cortex. Ultimately, the similarities between our year‐2 follow‐up findings and the existing literature reinforce the notion that alcohol sipping may be a relevant phenotype for predicting future alcohol involvement, with ties to reward and inhibitory control (Watts et al. 2021).

## Author Contributions

**Isabella F. Jackson:** conceptualization, data curation, formal analysis, methodology, software, validation, visualization, writing – original draft. **Francisco A. C. Meyer:** methodology, software, writing – review and editing. **Ryan M. Sullivan:** writing – review and editing. **Ashley L. Watts:** conceptualization, data curation, formal analysis, funding acquisition, methodology, project administration, resources, software, supervision, validation, visualization, writing – review and editing.

## Funding

I.F.J. and A.L.W. are supported by funding from the National Institutes of Health R00AA028306 (PI: Watts). R.M.S. was supported by NIDA (F32DA064409; PI: Sullivan).

## Conflicts of Interest

The authors declare no conflicts of interest.

## Supporting information


**Table S1:** Cortical structural volume across waves.
**Table S2:** Cortical MID large loss vs. neutral across waves.
**Table S3:** Cortical MID large reward vs. neutral across waves.
**Table S4:** Cortical ENB positive face vs. neutral across waves.
**Table S5:** Cortical ENB negative face vs. neutral across waves.
**Table S6:** Subcortical structural volume across waves.
**Table S7:** Subcortical MID large loss vs. neutral across waves.
**Table S8:** Subcortical MID large reward vs. neutral across waves.
**Table S9:** Subcortical ENB negative face vs. neutral across waves.
**Table S10:** Subcortical ENB positive face vs. neutral across waves.
**Table S11:** ICCs for cortical neuroimaging indices across waves.
**Table S12:** ICCs for subcortical neuroimaging indices across waves.
**Table S13:** Logistic regressions for inclusion odds of neuorimaging indices across waves.
**Table S14:** Cortical structural volume.
**Table S15:** Cortical MID large loss versus neutral.
**Table S16:** Cortical MID large reward versus neutral.
**Table S17:** ENB positive face versus neutral.
**Table S18:** ENB negative face versus neutral.
**Table S19:** Subcortical structural volume.
**Table S20:** Subcortical MID large loss vs. neutral.
**Table S21:** Subcortical MID large reward vs. neutral.
**Table S22:** Subcortical ENB negative face vs. neutral.
**Table S23:** Subcortical ENB positive face vs. neutral.
**Table S24:** Cortical structural volume.
**Table S25:** Cortical MID large loss vs. neutral.
**Table S26:** Cortical MID large reward vs. neutral.
**Table S27:** Cortical ENB positive face vs. neutral.
**Table S28:** Cortical ENB negative face vs. neutral.
**Table S29:** Subcortical structural volume.
**Table S30:** Subcortical MID large loss vs. neutral.
**Table S31:** Subcortical MID large reward vs. neutral.
**Table S32:** Subcortical ENB negative face vs. neutral.
**Table S33:** Subcortical ENB positive face vs. neutral.
**Table S34:** Cortical structural volume across waves.
**Table S35:** Cortical MID large loss vs neutral across waves.
**Table S36:** Cortical MID large reward vs neutral across waves.
**Table S37:** Cortical ENB positive face vs neutral across waves.
**Table S38:** Cortical ENB negative face vs neutral across waves.
**Table S39:** Subcortical structural volume across waves.
**Table S40:** Subcortical MID large loss vs neutral across waves.
**Table S41:** Subcortical MID large reward vs neutral across waves.
**Table S42:** Subcortical ENB negative face vs neutral across waves.
**Table S43:** Subcortical ENB positive face vs neutral across waves.

## Data Availability

Data used in the preparation of this article were obtained from the Adolescent Brain Cognitive Study (ABCD; https://abcdstudy.org), held in the NIMH Data Archive (NDA). This is a multisite, longitudinal study designed to recruit more than 10,000 children aged 9–10 and follow them over 10 years into early adulthood. The ABCD study is supported by the National Institutes of Health and additional federal partners under award numbers U01DA041048, U01DA050989, U01DA051016, U01DA041022, U01DA051018, U01DA051037, U01DA050987, U01DA041174, U01DA041106, U01DA041117, U01DA041028, U01DA041134, U01DA050988, U01DA051039, U01DA041156, U01DA041025, U01DA041120, U01DA051038, U01DA041148, U01DA041093, U01DA041089, U24DA041123, U24DA041147. A full list of supporters is available at https://abcdstudy.org/federal‐partners.html. A listing of participating sites and a complete listing of the study investigators can be found at https://abcdstudy.org/consortium_members/. ABCD consortium investigators designed and implemented the study and/or provided data but did not necessarily participate in the analysis or writing of this report. This manuscript reflects the views of the authors and may not reflect the opinions or views of the NIH or ABCD consortium investigators. The ABCD data repository grows and changes over time. The ABCD data used in this report came from DOI: 10.15154/8873‐zj65. The code necessary to reproduce the analyses presented here are publicly accessible: https://doi.org/10.17605/OSF.IO/NSHFB.

## References

[acer70370-bib-0001] Baranger, D. A. A. , S. E. Paul , A. S. Hatoum , and R. Bogdan . 2023. “Alcohol Use and Grey Matter Structure: Disentangling Predispositional and Causal Contributions in Human Studies.” Addiction Biology 28, no. 9: e13327. 10.1111/adb.13327. 37644894 PMC10502907

[acer70370-bib-0002] Bates, D. , M. Mächler , B. Bolker , and S. Walker . 2015. “Fitting Linear Mixed‐Effects Models Using lme4.” Journal of Statistical Software 67, no. 1: 1–48. 10.18637/jss.v067.i01.

[acer70370-bib-0003] Casey, B. J. , T. Cannonier , M. I. Conley , et al. 2018. “The Adolescent Brain Cognitive Development (ABCD) Study: Imaging Acquisition Across 21 Sites.” Developmental Cognitive Neuroscience 32: 43–54. 10.1016/j.dcn.2018.03.001. 29567376 PMC5999559

[acer70370-bib-0004] Cheetham, A. , N. B. Allen , S. Whittle , J. Simmons , M. Yücel , and D. I. Lubman . 2014. “Volumetric Differences in the Anterior Cingulate Cortex Prospectively Predict Alcohol‐Related Problems in Adolescence.” Psychopharmacology 231, no. 8: 1731–1742. 10.1007/s00213-014-3483-8. 24553579

[acer70370-bib-0005] Cohen, A. , M. Conley , D. Dellarco , and B. Casey . 2016. “The Impact of Emotional Cues on Short‐Term and Long‐Term Memory During Adolescence.” Proceedings of the Society for Neuroscience. San Diego, CA. November.

[acer70370-bib-0006] Cope, L. M. , M. E. Martz , J. E. Hardee , R. A. Zucker , and M. M. Heitzeg . 2019. “Reward Activation in Childhood Predicts Adolescent Substance Use Initiation in a High‐Risk Sample.” Drug and Alcohol Dependence 194: 318–325. 10.1016/j.drugalcdep.2018.11.003. 30471583 PMC6540995

[acer70370-bib-0007] Cservenka, A. 2016. “Neurobiological Phenotypes Associated With a Family History of Alcoholism.” Drug and Alcohol Dependence 158: 8–21. 10.1016/j.drugalcdep.2015.10.021. 26559000 PMC4698007

[acer70370-bib-0008] Cservenka, A. , and S. Azma . 2025. “Neural Correlates Associated With a Family History of Alcohol Use Disorder: A Narrative Review of Recent Findings.” Alcoholism, Clinical and Experimental Research 49, no. 1: 62–80. 10.1111/acer.15488. 39552054

[acer70370-bib-0009] Cservenka, A. , M. M. Herting , K. L. M. Seghete , K. A. Hudson , and B. J. Nagel . 2013. “High and Low Sensation Seeking Adolescents Show Distinct Patterns of Brain Activity During Reward Processing.” NeuroImage 66: 184–193. 10.1016/j.neuroimage.2012.11.003. 23142276 PMC3604176

[acer70370-bib-0010] Desikan, R. S. , F. Ségonne , B. Fischl , et al. 2006. “An Automated Labeling System for Subdividing the Human Cerebral Cortex on MRI Scans Into Gyral Based Regions of Interest.” NeuroImage 31, no. 3: 968–980. 10.1016/j.neuroimage.2006.01.021. 16530430

[acer70370-bib-0011] DeWit, D. J. , E. M. Adlaf , D. R. Offord , and A. C. Ogborne . 2000. “Age at First Alcohol Use: A Risk Factor for the Development of Alcohol Disorders.” American Journal of Psychiatry 157, no. 5: 745–750. 10.1176/appi.ajp.157.5.745. 10784467

[acer70370-bib-0012] Dichter, G. S. , R. V. Kozink , F. J. McClernon , and M. J. Smoski . 2012. “Remitted Major Depression Is Characterized by Reward Network Hyperactivation During Reward Anticipation and Hypoactivation During Reward Outcomes.” Journal of Affective Disorders 136, no. 3: 1126–1134. 10.1016/j.jad.2011.09.048. 22036801 PMC3272083

[acer70370-bib-0013] Donovan, J. E. , and B. S. G. Molina . 2008. “Children's Introduction to Alcohol Use: Sips and Tastes.” Alcoholism: Clinical and Experimental Research 32, no. 1: 108–119. 10.1111/j.1530-0277.2007.00565.x. 18070249 PMC2212613

[acer70370-bib-0014] Donovan, J. E. , and B. S. G. Molina . 2013. “Types of Alcohol Use Experience From Childhood Through Adolescence.” Journal of Adolescent Health 53, no. 4: 453–459. 10.1016/j.jadohealth.2013.03.024. PMC3783556 23763961

[acer70370-bib-0015] Feldstein Ewing, S. W. , A. Sakhardande , and S.‐J. Blakemore . 2014. “The Effect of Alcohol Consumption on the Adolescent Brain: A Systematic Review of MRI and fMRI Studies of Alcohol‐Using Youth.” NeuroImage: Clinical 5: 420–437. 10.1016/j.nicl.2014.06.011. 26958467 PMC4749850

[acer70370-bib-0016] Ferariu, A. , H. Chang , A. Taylor , and F. Zhang . 2024. “Alcohol Sipping Patterns, Personality, and Psychopathology in Children: Moderating Effects of Dorsal Anterior Cingulate Cortex (dACC) Activation.” Alcoholism, Clinical and Experimental Research 48, no. 8: 1492–1506. 10.1111/acer.15393. 38890123

[acer70370-bib-0017] Fischl, B. , D. H. Salat , E. Busa , et al. 2002. “Whole Brain Segmentation.” Neuron 33, no. 3: 341–355. 10.1016/S0896-6273(02)00569-X. 11832223

[acer70370-bib-0018] Garavan, H. , H. Bartsch , K. Conway , et al. 2018. “Recruiting the ABCD Sample: Design Considerations and Procedures.” Developmental Cognitive Neuroscience 32: 16–22. 29703560 10.1016/j.dcn.2018.04.004 PMC6314286

[acer70370-bib-0019] Gasquoine, P. G. 2014. “Contributions of the Insula to Cognition and Emotion.” Neuropsychology Review 24, no. 2: 77–87. 10.1007/s11065-014-9246-9. 24442602

[acer70370-bib-0020] Gonçalves, P. D. , S. S. Martins , N. M. Gebru , et al. 2024. “Associations Between Family History of Alcohol and/or Substance Use Problems and Frontal Cortical Development From 9 to 13 Years of Age: A Longitudinal Analysis of the ABCD Study.” Biological Psychiatry Global Open Science 4, no. 2: 100284. 10.1016/j.bpsgos.2023.100284. 38312852 PMC10837483

[acer70370-bib-0021] Haber, S. N. , and B. Knutson . 2010. “The Reward Circuit: Linking Primate Anatomy and Human Imaging.” Neuropsychopharmacology 35, no. 1: 4–26. 10.1038/npp.2009.129. 19812543 PMC3055449

[acer70370-bib-0022] Hagler, D. J., Jr. , S. Hatton , M. D. Cornejo , et al. 2019. “Image Processing and Analysis Methods for the Adolescent Brain Cognitive Development Study.” NeuroImage 202: 116091. 10.1016/j.neuroimage.2019.116091. 31415884 PMC6981278

[acer70370-bib-0023] Harris, J. C. , M. T. Liuzzi , B. A. Malames , C. L. Larson , and K. M. Lisdahl . 2023. “Differences in Parent and Youth Perceived Neighborhood Threat on Nucleus Accumbens‐Frontoparietal Network Resting State Connectivity and Alcohol Sipping in Children Enrolled in the ABCD Study.” Frontiers in Psychiatry 14: 1237163. 10.3389/fpsyt.2023.1237163. 37928910 PMC10622767

[acer70370-bib-0024] Hawkins, J. D. , J. W. Graham , E. Maguin , R. Abbott , K. G. Hill , and R. F. Catalano . 1997. “Exploring the Effects of Age of Alcohol Use Initiation and Psychosocial Risk Factors on Subsequent Alcohol Misuse.” Journal of Studies on Alcohol 58, no. 3: 280–290. 10.15288/jsa.1997.58.280. 9130220 PMC1894758

[acer70370-bib-0025] Herlin, B. , V. Navarro , and S. Dupont . 2021. “The Temporal Pole: From Anatomy to Function—A Literature Appraisal.” Journal of Chemical Neuroanatomy 113: 101925. 10.1016/j.jchemneu.2021.101925. 33582250

[acer70370-bib-0026] Herting, M. M. , P. Gautam , Z. Chen , A. Mezher , and N. C. Vetter . 2018. “Test‐Retest Reliability of Longitudinal Task‐Based fMRI: Implications for Developmental Studies.” Developmental Cognitive Neuroscience 33: 17–26. 10.1016/j.dcn.2017.07.001. 29158072 PMC5767156

[acer70370-bib-0027] Hingson, R. W. , T. Heeren , and M. R. Winter . 2006. “Age at Drinking Onset and Alcohol Dependence: Age at Onset, Duration, and Severity.” Archives of Pediatrics & Adolescent Medicine 160, no. 7: 739. 10.1001/archpedi.160.7.739. 16818840

[acer70370-bib-0028] Hooker, C. I. , and R. T. Knight . 2006. “The Role of Lateral Orbitofrontal Cortex in the Inhibitory Control of Emotion.” In The Orbitofrontal Cortex, vol. 307, 1–18. Oxford University Press.

[acer70370-bib-0029] Jackson, K. M. , N. P. Barnett , S. M. Colby , and M. L. Rogers . 2015. “The Prospective Association Between Sipping Alcohol by the Sixth Grade and Later Substance Use.” Journal of Studies on Alcohol and Drugs 76, no. 2: 212–221. 10.15288/jsad.2015.76.212. 25785796 PMC5374474

[acer70370-bib-0030] Jacobus, J. , and S. F. Tapert . 2013. “Neurotoxic Effects of Alcohol in Adolescence.” Annual Review of Clinical Psychology 9, no. 1: 703–721. 10.1146/annurev-clinpsy-050212-185610. PMC3873326 23245341

[acer70370-bib-0031] Johnston, L. , R. Miech , P. O'Malley , J. Bachman , J. Schulenberg , and M. Patrick . 2020. “Monitoring the Future National Survey Results on Drug Use, 1975–2019: Overview, Key Findings on Adolescent Drug Use.”

[acer70370-bib-0032] Kirsch, D. , C. M. Nemeroff , and E. T. C. Lippard . 2020. “Early Life Stress and Substance Use Disorders: Underlying Neurobiology and Pathways to Adverse Outcomes.” Adversity and Resilience Science 1, no. 1: 29–47. 10.1007/s42844-020-00005-7. PMC11995910 40235604

[acer70370-bib-0033] Knutson, B. , A. Westdorp , E. Kaiser , and D. Hommer . 2000. “FMRI Visualization of Brain Activity During a Monetary Incentive Delay Task.” NeuroImage 12, no. 1: 20–27. 10.1006/nimg.2000.0593. 10875899

[acer70370-bib-0034] Kwarteng, A. E. , M. M. Rahman , D. G. Gee , M. A. Infante , S. F. Tapert , and B. L. Curtis . 2021. “Child Reward Neurocircuitry and Parental Substance Use History: Findings From the Adolescent Brain Cognitive Development Study.” Addictive Behaviors 122: 107034. 10.1016/j.addbeh.2021.107034. 34246036 PMC8328938

[acer70370-bib-0035] Lees, B. , L. Aguinaldo , L. M. Squeglia , et al. 2020. “Parental Family History of Alcohol Use Disorder and Neural Correlates of Response Inhibition in Children From the Adolescent Brain Cognitive Development (ABCD) Study.” Alcoholism: Clinical and Experimental Research 44, no. 6: 1234–1244. 10.1111/acer.14343. 32333792 PMC7323844

[acer70370-bib-0036] Lees, B. , L. Mewton , L. A. Stapinski , L. M. Squeglia , C. D. Rae , and M. Teesson . 2019. “Neurobiological and Cognitive Profile of Young Binge Drinkers: A Systematic Review and Meta‐Analysis.” Neuropsychology Review 29, no. 3: 357–385. 10.1007/s11065-019-09411-w. 31512192 PMC7231524

[acer70370-bib-0037] Lenth, R. V. 2016. “Least‐Squares Means: The R Package Lsmeans.” Journal of Statistical Software 69, no. 1: 1–33. 10.18637/jss.v069.i01.

[acer70370-bib-0038] Lisdahl, K. M. , S. Tapert , K. J. Sher , et al. 2021. “Substance Use Patterns in 9–10 Year Olds: Baseline Findings From the Adolescent Brain Cognitive Development (ABCD) Study.” Drug and Alcohol Dependence 227: 108946. 10.1016/j.drugalcdep.2021.108946. 34392051 PMC8833837

[acer70370-bib-0039] Lüdecke, D. , M. S. Ben‐Shachar , I. Patil , and D. Makowski . 2020. “Extracting, Computing and Exploring the Parameters of Statistical Models Using R.” Journal of Open Source Software 5, no. 53: 2445. 10.21105/joss.02445.

[acer70370-bib-0040] Makris, N. , M. Oscar‐Berman , S. K. Jaffin , et al. 2008. “Decreased Volume of the Brain Reward System in Alcoholism.” Biological Psychiatry 64, no. 3: 192–202. 10.1016/j.biopsych.2008.01.018. 18374900 PMC2572710

[acer70370-bib-0041] Marek, S. , B. Tervo‐Clemmens , F. J. Calabro , et al. 2022. “Reproducible Brain‐Wide Association Studies Require Thousands of Individuals.” Nature 603, no. 7902: 654–660. 10.1038/s41586-022-04492-9. 35296861 PMC8991999

[acer70370-bib-0042] Marshall, E. J. 2014. “Adolescent Alcohol Use: Risks and Consequences.” Alcohol and Alcoholism 49, no. 2: 160–164. 24402246 10.1093/alcalc/agt180

[acer70370-bib-0043] Martz, M. E. , J. E. Hardee , L. M. Cope , et al. 2022. “Nucleus Accumbens Response to Reward Among Children With a Family History of Alcohol Use Problems: Convergent Findings From the ABCD Study and Michigan Longitudinal Study.” Brain Sciences 12, no. 7: 913. 35884720 10.3390/brainsci12070913 PMC9320357

[acer70370-bib-0044] Maurage, P. , P. E. G. Bestelmeyer , J. Rouger , I. Charest , and P. Belin . 2013. “Binge Drinking Influences the Cerebral Processing of Vocal Affective Bursts in Young Adults.” NeuroImage: Clinical 3: 218–225. 10.1016/j.nicl.2013.08.010. 24179866 PMC3791281

[acer70370-bib-0045] May, A. C. , J. Jacobus , A. N. Simmons , and S. F. Tapert . 2022. “A Prospective Investigation of Youth Alcohol Experimentation and Reward Responsivity in the ABCD Study.” Frontiers in Psychiatry 13: 886848. 10.3389/fpsyt.2022.886848. 36003980 PMC9393480

[acer70370-bib-0046] Morales, A. M. , S. A. Jones , A. Ehlers , J. B. Lavine , and B. J. Nagel . 2018. “Ventral Striatal Response During Decision Making Involving Risk and Reward Is Associated With Future Binge Drinking in Adolescents.” Neuropsychopharmacology 43, no. 9: 1884–1890. 10.1038/s41386-018-0087-8. 29789576 PMC6046057

[acer70370-bib-0047] Nguyen‐Louie, T. T. , A. N. Simmons , L. M. Squeglia , M. Alejandra Infante , J. P. Schacht , and S. F. Tapert . 2018. “Earlier Alcohol Use Onset Prospectively Predicts Changes in Functional Connectivity.” Psychopharmacology 235, no. 4: 1041–1054. 10.1007/s00213-017-4821-4. 29306963 PMC5871543

[acer70370-bib-0048] O'Halloran, L. , C. Nymberg , L. Jollans , H. Garavan , and R. Whelan . 2017. “The Potential of Neuroimaging for Identifying Predictors of Adolescent Alcohol Use Initiation and Misuse.” Addiction 112, no. 4: 719–726. 10.1111/add.13629. 27917536

[acer70370-bib-0049] Pérez‐García, J. M. , S. Suárez‐Suárez , S. Doallo , and F. Cadaveira . 2022. “Effects of Binge Drinking During Adolescence and Emerging Adulthood on the Brain: A Systematic Review of Neuroimaging Studies.” Neuroscience & Biobehavioral Reviews 137: 104637. 10.1016/j.neubiorev.2022.104637. 35339481

[acer70370-bib-0050] R Core Team . 2024. R: A Language and Environment for Statistical Computing. R Foundation for Statistical Computing. https://www.R‐project.org/.

[acer70370-bib-0051] Rolls, E. T. , W. Cheng , and J. Feng . 2020. “The Orbitofrontal Cortex: Reward, Emotion and Depression.” Brain Communications 2, no. 2: fcaa196. 10.1093/braincomms/fcaa196. 33364600 PMC7749795

[acer70370-bib-0052] Seo, S. , A. Beck , C. Matthis , et al. 2019. “Risk Profiles for Heavy Drinking in Adolescence: Differential Effects of Gender.” Addiction Biology 24, no. 4: 787–801. 10.1111/adb.12636. 29847018

[acer70370-bib-0053] Squeglia, L. M. , J. Jacobus , and S. F. Tapert . 2014. “Chapter 28—The Effect of Alcohol Use on Human Adolescent Brain Structures and Systems.” In Alcohol and the Nervous System, edited by E. V. Sullivan and A. Pfefferbaum , vol. 125, 501–510. Elsevier. 10.1016/B978-0-444-62619-6.00028-8. PMC4321715 25307592

[acer70370-bib-0054] Squeglia, L. M. , D. A. Rinker , H. Bartsch , et al. 2014. “Brain Volume Reductions in Adolescent Heavy Drinkers.” Developmental Cognitive Neuroscience 9: 117–125. 10.1016/j.dcn.2014.02.005. 24632141 PMC4061267

[acer70370-bib-0055] Swick, D. , V. Ashley , and A. U. Turken . 2008. “Left Inferior Frontal Gyrus Is Critical for Response Inhibition.” BMC Neuroscience 9, no. 1: 102. 10.1186/1471-2202-9-102. 18939997 PMC2588614

[acer70370-bib-0056] Uddin, L. Q. 2015. “Salience Processing and Insular Cortical Function and Dysfunction.” Nature Reviews Neuroscience 16, no. 1: 55–61. 10.1038/nrn3857. 25406711

[acer70370-bib-0057] Van Den Heuvel, M. P. , and H. E. Hulshoff Pol . 2010. “Exploring the Brain Network: A Review on Resting‐State fMRI Functional Connectivity.” European Neuropsychopharmacology 20, no. 8: 519–534. 10.1016/j.euroneuro.2010.03.008. 20471808

[acer70370-bib-0058] Vijayakumar, N. , N. B. Allen , G. Youssef , et al. 2016. “Brain Development During Adolescence: A Mixed‐Longitudinal Investigation of Cortical Thickness, Surface Area, and Volume.” Human Brain Mapping 37, no. 6: 2027–2038. 10.1002/hbm.23154. 26946457 PMC6867680

[acer70370-bib-0059] Waller, R. , L. Murray , D. S. Shaw , E. E. Forbes , and L. W. Hyde . 2019. “Accelerated Alcohol Use Across Adolescence Predicts Early Adult Symptoms of Alcohol Use Disorder via Reward‐Related Neural Function.” Psychological Medicine 49, no. 4: 675–684. 10.1017/S003329171800137X. 29871712 PMC7066874

[acer70370-bib-0060] Watts, A. L. , M. I. Doss , D. L. Bernard , and K. J. Sher . 2024. “Psychopathology as Dynamic Markers of Alcohol Initiation Across Development: A Three‐Year Longitudinal Examination.” Development and Psychopathology 36, no. 2: 919–928. 10.1017/S0954579423000184. 36939078 PMC10509330

[acer70370-bib-0061] Watts, A. L. , P. K. Wood , K. M. Jackson , et al. 2021. “Incipient Alcohol Use in Childhood: Early Alcohol Sipping and Its Relations With Psychopathology and Personality.” Development and Psychopathology 33, no. 4: 1338–1350. 10.1017/S0954579420000541. 32522303 PMC7814694

[acer70370-bib-0062] Whelan, R. , R. Watts , C. A. Orr , et al. 2014. “Neuropsychosocial Profiles of Current and Future Adolescent Alcohol Misusers.” Nature 512, no. 7513: 185–189. 10.1038/nature13402. 25043041 PMC4486207

[acer70370-bib-0063] Whittle, S. , N. B. Allen , D. I. Lubman , and M. Yücel . 2006. “The Neurobiological Basis of Temperament: Towards a Better Understanding of Psychopathology.” Neuroscience & Biobehavioral Reviews 30, no. 4: 511–525. 10.1016/j.neubiorev.2005.09.003. 16289282

[acer70370-bib-0064] Yarkoni, T. , R. A. Poldrack , T. E. Nichols , D. C. Van Essen , and T. D. Wager . 2011. “Large‐Scale Automated Synthesis of Human Functional Neuroimaging Data.” Nature Methods 8, no. 8: 665–670. 21706013 10.1038/nmeth.1635 PMC3146590

[acer70370-bib-0065] Zeng, J. , L. You , F. Yang , et al. 2023. “A Meta‐Analysis of the Neural Substrates of Monetary Reward Anticipation and Outcome in Alcohol Use Disorder.” Human Brain Mapping 44, no. 7: 2841–2861. 10.1002/hbm.26249. 36852619 PMC10089105

[acer70370-bib-0066] Zhang, W.‐N. , S.‐H. Chang , L.‐Y. Guo , K.‐L. Zhang , and J. Wang . 2013. “The Neural Correlates of Reward‐Related Processing in Major Depressive Disorder: A Meta‐Analysis of Functional Magnetic Resonance Imaging Studies.” Journal of Affective Disorders 151, no. 2: 531–539. 10.1016/j.jad.2013.06.039. 23856280

